# Intestinal-derived ILCs migrating in lymph increase IFNγ production in response to *Salmonella* Typhimurium infection

**DOI:** 10.1038/s41385-020-00366-3

**Published:** 2021-01-07

**Authors:** Verena Kästele, Johannes Mayer, Edward S. Lee, Natalie Papazian, John J. Cole, Vuk Cerovic, Gabrielle Belz, Michio Tomura, Gerard Eberl, Carl Goodyear, Rose A. Maciewicz, Daniel Wall, Tom Cupedo, David R. Withers, Simon Milling

**Affiliations:** 1 Institute of Infection, Immunity and Inflammation, University of Glasgow, Glasgow, UK; 2 Department of Pathology, Albert Einstein College of Medicine, Bronx, NY USA; 3 Department of Haematology, Erasmus University Medical Center, Rotterdam, The Netherlands; 4 Walter+Eliza Hall Institute of Medical Research, Parkville, VIC Australia; 5 Institute of Molecular Medicine, University Hospital, RWTH Aachen, Aachen, Germany; 6 Osaka Ohtani University, Osaka, Japan; 7 Microenvironment & Immunity Unit, Institut Pasteur, Paris, France; 8 Institute of Immunology & Immunotherapy, College of Medical and Dental Sciences, University of Birmingham, Birmingham, UK

## Abstract

Innate lymphoid cells (ILCs) are enriched in mucosae and have been described as tissue-resident. Interestingly, ILCs are also present within lymph nodes (LNs), in the interfollicular regions, the destination for lymph-migratory cells. We have previously shown that LN ILCs are supplemented by peripheral tissue-derived ILCs. Using thoracic duct cannulations, we here enumerate the intestinal lymph ILCs that traffic from the intestine to the mesenteric LNs (MLNs). We provide, for the first time, a detailed characterisation of these lymph-migratory ILCs. We show that all ILC subsets migrate in lymph, and while global transcriptional analysis reveals a shared signature with tissue-resident ILCs, lymph ILCs express migration-associated genes including *S1PR*s, *SELL* (CD62L) and *CCR7*. Interestingly, we discovered that while *Salmonella* Typhimurium infections do not increase the numbers of migrating ILCs, infection changes their composition and cytokine profile. Infection increases the proportions of RORyt^+^ T-bet^+^ ILCs, levels of IFNγ, and IFNγ/GM-CSF co-expression. Infection-induced changes in migratory ILCs are reflected in colon-draining MLN ILCs, where RORyt^+^ T-bet^+^ ILCs accumulate and display corresponding increased cytokine expression. Thus, we reveal that ILCs respond rapidly to intestinal infection and can migrate to the MLN where they produce cytokines.

## Introduction

Innate lymphoid cells (ILCs), are present throughout the body and make important contributions to tissue homoeostasis and immunity. ILCs comprise three subsets termed ILC1, ILC2 and ILC3, distinguished by their transcription factor profile and effector cytokines.^1–3^ ILCs are distinct from NK cells and are collectively termed ‘helper’ ILCs due to a lack of cytotoxic function and identified by their common expression of IL-7Rα.^2,4,5^ These helper ILCs are enriched at barrier sites including the gastrointestinal tract, where they are described as tissue-resident populations.^6–8^ However, in human blood, whilst progenitor populations are found, ILCs comparable to ‘mature’ tissue ILCs have also been described.^1,2,9,10^ Furthermore. trafficking of ILCs from the intestine to the MLN has been demonstrated, showing that not all murine ILCs remain anchored in tissues.^11^ Therefore, secondary lymphoid tissues contain migratory ILC populations, potentially enabling tissue-derived ILCs to influence lymph node immune responses.

Dysregulation of ILC functions contributes to pathology in inflammatory diseases, particularly at barrier sites.^12–14^ While lymphoid tissue inducer cells (LTi), an ILC3 population, are required for lymphoid organogenesis, the contributions of ILCs to adaptive immunity in secondary lymphoid tissues are less well understood. Although there is clear evidence for ILC regulation of adaptive immunity,^15–17^ questions remain about where these interactions occur and the contributions of tissue-derived ILCs.

In the MLNs, ILC2 and ILC3 are located in the interfollicular area of the LN, where lymph enters the node. Migratory cells such as dendritic cells (DCs) pass through this area.^11,18^ Because of the location of ILCs in MLN, combined with their migratory capacity, we hypothesised that lymph-migratory ILCs might be equipped to influence the initiation of adaptive immune responses. To test this, we assessed migratory cells in lymph isolated by thoracic duct cannulations of either intact mice, or mice that had undergone mesenteric lymphadenectomy. Here we provide an extended description of these intestinal migratory ILCs in health and after an acute infection. Interestingly, migratory ILCs change their composition and transcriptomic profile after *Salmonella* Typhimurium (STM) infection. In infected mice we find more migratory RORyt^+^ T-bet^+^ ILCs, with increased levels of IFNγ and some co-expression of GM-CSF. Similar cytokine and transcription factor profile changes are observed in the colon-draining LN. Thus, migratory ILCs respond to their environment, travel to the LNs, and are equipped to contribute to immune responses therein.

## Results

### A population of ILCs continuously migrates in intestinal lymph

**Fig. 1:**
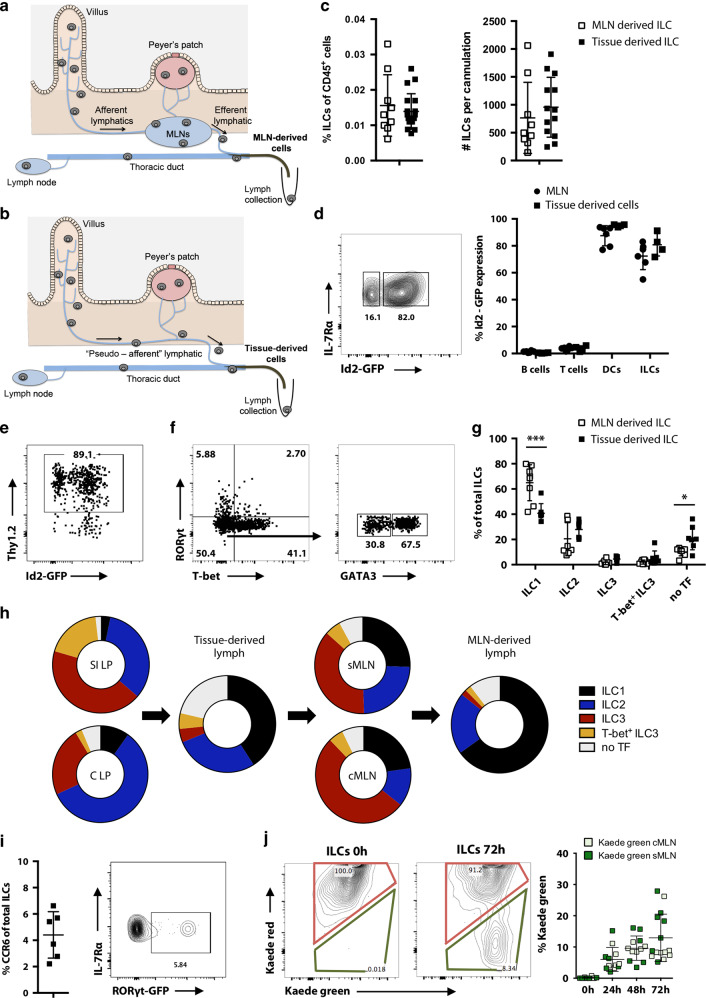
A population of ILCs continuously migrates in intestinal lymph. Schematic figures of the lymphatic system in the small intestine of (**a**) intact and (**b**) mesenteric lymphadenectomised mice are shown. Lymph and migratory cells were isolated by thoracic duct cannulation of intact (MLN-derived lymph) or mesenteric lymphadenectomised (tissue-derived lymph) C57BL/6 mice and collected for 18–24 h. **c** ILCs in tissue-derived and MLN-derived lymph were identified as live, single, CD45^+^ IL-7Rα^+^ lineage^−^ (CD11b^−^ CD11c^−^ B220^−^ CD3^−^ CD5^−^ intracellular CD3^−^) cells. The percentage and total cell numbers of ILCs per cannulation were assessed by flow cytometry of at least three independent experiments (*n* = 9–19). **d** Migratory ILCs were analyzed for Id2 expression by cannulating MLNxed Id2^GFP/+^ reporter mice. Additionally, cells were isolated from mesenteric lymph nodes (MLNs) of age matched Id2^GFP/+^ reporter mice. A representative FACS plot for Id2 expression in lymph ILCs (CD45^+^ IL-7Ra^+^ lineage^−^ cells) and percentages of Id2 expression in MLNs and lymph ILCs, B cells (live, single, CD45^+^ B220^+^ cells), T cells (live, single, CD45^+^ CD3^+^ CD5^+^ cells) and migratory DCs (live, single, CD45^+^ MHCII^high^ CD11c^+^ CD64^−^ cells) are shown. Data from two independent experiments for MLN and three independent experiments for lymph samples are shown. Each dot represents an individual animal (*n* = 4–6). **e** FACS plot of Thy1.2 expression on migratory ILCs isolated from afferent lymph of Id2^GFP/+^ mice is shown. **f** Plot shows tissue-derived cells isolated by thoracic duct cannulations of C57BL/6 mice that had undergone mesenteric lymphadenectomy and ILC subsets were identified by staining for the transcription factors T-bet, GATA3 and RORγt. **g** MLN-derived cells and tissue-derived cells were isolated by thoracic duct cannulation and the composition of ILCs assessed. Data are representative for at least three independent experiments (*n* = 7–8). Each dot represents an individual animal. **h** Proportions of each type of ILCs in each tissue compartment are represented by donut plots. Each donut represents data from at least two independent experiments (*n* = 6–8). **i** Percentages of CCR6 expressing ILCs in tissue-derived lymph of C57BL/6 mice are displayed. Data from three independent experiments are shown (*n* = 6). FACS plot of RORyt expression in tissue-derived ILCs in a *Rorc(γt)*^+/GFP^ reporter mouse that previously underwent mesenteric lymphadenectomy is shown. **j** At laparotomy, MLNs of Kaede mice were exposed to low-intensity violet light. Small intestinal (sMLN) or colonic (cMLN) draining lymph nodes were removed either immediately or after 24, 48 or 72 h. Representative FACS plots of photoconverted Kaede red ILCs (live, single, CD45^+^ IL-7Rα^+^ lineage^−^ Kaede green^+^ Kaede red^+^) and non-converted Kaede green ILCs (live, single, CD45^+^ IL-7Rα^+^ lineage^−^ Kaede green^+^ Kaede red^−^) in sMLNs at 0 and 72 h after laparotomy are shown. Graph displays the expression of non-converted Kaede green ILCs in the sMLNs (dark colour) or cMLNs (light colour). Each dot represents an individual animal with sMLNs and cMLNs harvested from the same animal (*n* = 5–8) in two independent experiments.

ILCs at barrier sites have been mainly described as tissue-resident cells, including in the gastrointestinal tract.^6^ Interactions of ILCs with other immune cells are important for regulating adaptive immune responses.^15,19,20^ The importance of ILCs in LNs has recently been highlighted by reports that they contribute to the regulation of commensal-specific T cells and IgA production.^15,16^ We previously reported that some ILCs in the MLNs originate from the intestine^11^ but much remains unknown, including a detailed description of numbers and phenotype of migratory ILCs in steady state and during inflammation. To assess whether ILCs traffic to LNs via lymphatic vessels we characterised migratory cells in afferent lymph, collected by thoracic duct cannulations. Lymph was collected from both intact and mesenteric lymphadenectomised (MLNx) mice.^21^ Cannulation of intact mice allows the collection of cells that have exited the MLNs via the efferent lymphatics (MLN-derived cells) (Fig. 1a), whereas pseudo-afferent lymph from MLNx mice contains cells that have exited the intestine (tissue-derived cells) (Fig. 1b). After cannulation, lymph is collected from conscious freely moving animals for 18–24 h. We analysed the cellular composition of intestine-derived lymph by staining for CD4 and CD8 T cells (CD45^+^ CD3^+^ CD5^+^ CD4^+^ or CD8^+^), B cells (CD45^+^ B220^+^), dendritic cells (DCs) (CD45^+^ MHCII^+^ CD11c^+^) and ILCs (CD45^+^, CD3^−^, CD5^−^, B220^−^, CD11c^−^, CD11b^−^, IL-7Ra^+^, intracellular CD3^−^) (gating strategy Fig. S1A, B). The majority of intestinal migratory cells are B cells (60%), CD4^+^ T cells, (20%) and CD8^+^ T cells (20%). DCs comprise 1% of migratory cells^21^ (Fig. S1C). In both intestine- and MLN-derived lymph we identified a small but consistent ILC population. Both intestine- and MLN-derived lymph contain comparable frequencies of migratory ILCs; 0.01–0.03% of CD45^+^ cells. This represents ~200–2000 ILCs migrating per 24 h (Fig. 1c). To confirm that these cells belong to the ILC family, we assessed their expression of Id2, a key transcription factor for ILC lineage specificity.^22^ We examined Id2-GFP expression in cells from intestine-derived lymph and MLNs of Id2^GFP/+^ mice.^23^ Whereas B cells and T cells lack Id2 expression, 70–95% of DCs and ILCs in both afferent lymph and MLNs were Id2 positive. Frequencies of Id2^+^ ILCs in the lymph are comparable to frequencies in the MLN (Fig. 1d). Although 10–20% of lymph-migratory ILCs appear Id2 negative, they express Thy1.2 (Fig. 1e), indicating that they likely belong to the ILC family.^24^

We analysed the subset composition of migratory ILCs by staining for T-bet, GATA3 and RORyt. In tissue-derived lymph, ILC1s (T-bet^+^, 40%) and ILC2s (GATA3^+^, 28%) were the most frequent among total migratory ILCs (Fig. 1f, g). Interestingly, although all ILCs subsets were also found in MLN-derived lymph, the majority of ILCs exiting the MLNs (65%) are T-bet positive ILC1s (Fig. 1g). Given that the composition of ILCs entering and leaving the MLN is different, some ILCs appear not to leave the MLN after having migrated from the intestine. Figure 1h shows the proportions of each ILC type in the different anatomical compartments. Although the majority of ILCs in the MLNs are ILC3s, as previously reported,^11^ few ILC3s appear to migrate in the lymph. Conversely, ILC1s were found at the highest frequencies in both intestine-derived and MLN-derived lymph. To confirm that few ILC3s migrate from the intestine, intestine-derived lymph ILCs were stained for CCR6, and were also collected from a *Rorc(γt)*^+/GFP^ mouse. Both CCR6 and RORγt-GFP signals were detected at low frequencies in intestine-derived lymph ILCs (~5% of total lymph ILCs) (Fig. 1i). Together, these data demonstrate that all intestinal ILC types migrate in lymph, although at different frequencies, and some of these ILCs are retained in the LN.

### ILCs migrate to MLNs from both the small intestine and colon

To address migratory intestinal ILCs dynamics in more detail, we assessed whether ILCs from both the colon and the small intestine migrate to the MLNs. We exposed MLNs of photoconvertible Kaede mice to violet light.^25^ Kaede mice ubiquitously express the Kaede green protein, which switches to Kaede red when exposed to violet light. Using low-intensity violet LED lights, MLNs were photoconverted while other tissues were light-protected to allow tissue-specific photoconversion. Because lymphatic vessels of the small intestine and the colon drain to different nodes within the chain of MLNs,^26^ we separately analysed colon draining (cMLN) and small intestine-draining MLN (sMLN), either immediately or 24, 48, or 72 h after MLN photoconversion. Cells located in MLNs at the time of photoconversion express both the Kaede green and the Kaede red proteins, whereas incoming migratory cells only express Kaede green. We can therefore distinguish recently migrated ILCs as Kaede green cells that lack the Kaede red. All MLN ILCs expressed photoconverted Kaede red protein when analysed immediately after photoconversion and no Kaede green single positive cells are detectable (Fig. 1j). The frequency of Kaede green-only migratory ILCs increases gradually up to 13% of total MLN ILCs by 72 h post photoconversion (Fig. 1j). In contrast, the majority of T cells have been replaced by incoming circulatory T cells after only 24 h (70% Kaede green of total T cells) (Fig. S1D). We detected similar frequencies of new incoming ILCs in both the sMLN and cMLNs, (Fig. 1j). In addition, lower frequencies of CCR6^+^ ILCs are found, both in the incoming Kaede green ILCs and in the lymph, than in Kaede red MLN-resident ILCs (Fig. S1E). Thus, migratory ILCs do not become CCR6^+^ after reaching the MLN. In summary, these data indicate that ILCs in the cMLN and sMLN are supplemented at similar proportions by migratory ILCs.

### Increased migration of colonic RORyt^+^ T-bet^+^ ILCs during *S*. Typhimurium infections

**Fig. 2:**
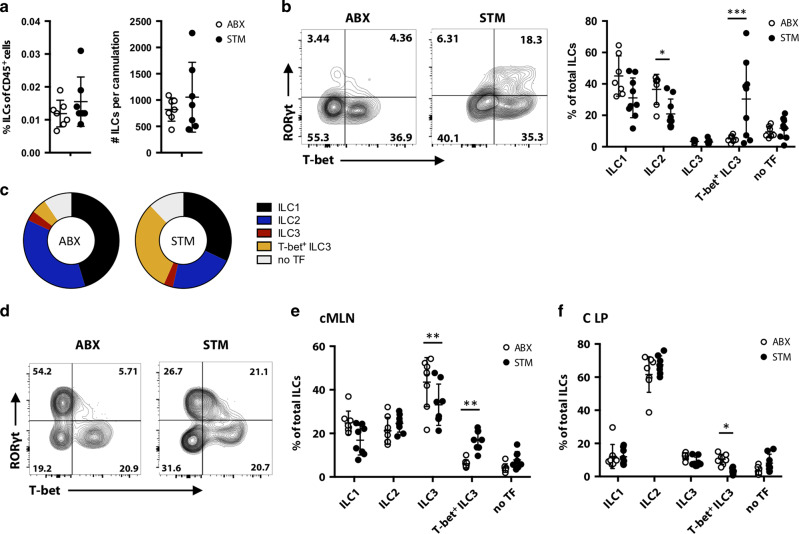
Increased migrating colonic RORyt^+^ T-bet^+^ ILCs after *S*. Typhimurium infection. C57BL/6 mice that previously underwent mesenteric lymphadenectomy received streptomycin (ABX) or received streptomycin and were infected orally after 24 h with *S*. Typhimurium (STM). Migratory cells were collected by thoracic duct cannulations 48 h post infection and cells collected for 18–24 h. Migratory cells were stained with antibodies for phenotypic analysis by flow cytometry. **a** Graphs show the percentages and total cell numbers of migratory ILCs per cannulation in lymph of *Salmonella* (STM) infected and control (ABX) mice. Each dot represents an individual animal (*n* = 7) from at least three independent experiments. **b** ILC subsets were analysed by assessing the expression of the transcription factors T-bet, GATA3 and RORγt. Representative FACS plots of T-bet and RORγt expression in migratory ILCs of STM and control mice are displayed. Graph shows the percentages of ILC subsets in control an STM-infected mice. Each dot represents an individual animal (*n* = 7–9) from at least three independent experiments. **c** Donut plots display the proportions of ILCs from control or STM-infected mice, Data from at least three independent experiments are shown (*n* = 7–9). **d** Intact C57BL/6 mice received streptomycin (ABX) or received streptomycin and were infected after 24 h with STM. Cells from colonic LP (C LP) and cMLNs were isolated 3 days post STM infection. ILC subsets were identified by the expression of the transcription T-bet, GATA3 and RORγt. **d** Representative FACS plots of T-bet and RORγt stained ILCs in cMLNs of STM infected and control mice are shown. Graph in (**e**) shows the composition of ILCs in the cMLN. **f** Shows ILC populations in the C LP of STM-infected and control (ABX) mice. Each dot represents an individual animal (*n* = 8) from at least three independent experiments.

We next assessed how intestinal infection impacts ILC migration. Therefore, we infected mice with STM, after pre-treatment with streptomycin.^27^ We chose STM infection because it provides a model of acute colonic inflammation, with reported contributions of ILCs in the early phase of the immune response.^28^ Consistent with the infection being localised primarily in the colon, we detected increased total cell numbers in the colonic lamina propria and the cMLN, but not the small intestine or sMLN (Fig. S1F). Because ILCs migrate from the colon to the cMLN, we assessed whether infection increased the numbers of migratory ILC in intestine-derived lymph. Thus, MLNx mice were infected with STM and intestinal lymph was isolated 48 h post infection. Control mice received streptomycin antibiotics (ABX) without infection. Staining of lymph revealed that STM infection does not alter the frequency or number of migratory ILCs in lymph (Fig. 2a). However, intestine-draining lymph of STM-infected mice showed an increased proportion of RORyt^+^ T-bet^+^ ILCs (labelled as T-bet^+^ ILC3s in figures), which are almost absent from lymph of control mice (Figs. 1h and 2b). The changes in ILC frequencies that occur in lymph after infection are summarised in Fig. 2c. Corresponding to the changes in intestinal lymph, we detected an accumulation of RORyt^+^ T-bet^+^ ILCs in the cMLN (Fig. 2d, e). The increased proportion of RORyt^+^ T-bet^+^ ILCs in the lymph and MLN after STM infection reflects changes in the colonic lamina propria, where this population was decreased after STM infection (Fig. 2f). We detected no differences in total ILC cell numbers or frequencies of RORyt^+^ T-bet^+^ ILCs in the SI LP or sMLN (Fig. S1G, H). Thus, following STM infection, changes in the composition of ILC subsets were restricted to the colon and cMLNs, and corresponding changes were observed in the migratory ILC population.

### The transcriptomic profile of ILCs in lymph closely matches with tissue-resident ILCs

**Fig. 3:**
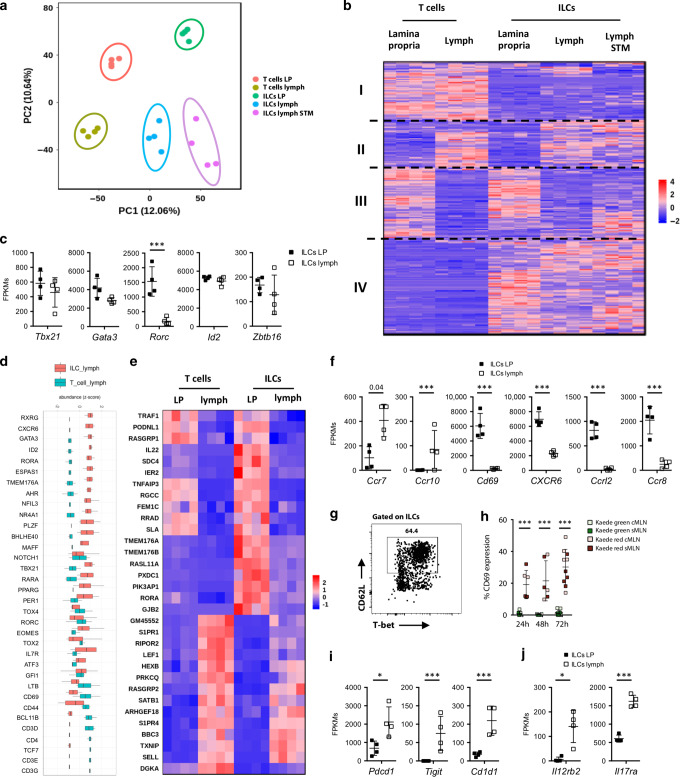
ILCs in lymph have a migratory gene expression signature. Cells from the lamina propria (LP) or lymph were isolated from C57BL/6 mice that previously underwent complete mesenteric lymphadenectomy. Lymph was isolated by thoracic duct cannulations and migratory cells were collected for 18–24 h. **a** Principal component analysis of gene expression data for ILCs and T cells isolated from the LP and lymph in steady state or after *STM* infection. The first two components are shown with replicates represented by dots. The total variation by each component is shown. **b** Heatmap shows the significantly different genes (fold change >2 and adjusted *p* value < 0.01) between T cells and ILCs isolated from the lamina propria and lymph in steady state and after STM infection. Genes (*y*-axis) and samples (*x*-axis) have been clustered using spearman distances and UPGMA agglomeration. **c** Transcription levels of selected genes in ILCs isolated from lymph compared to the lamina propria in steady state are displayed as FPKMs. **d** Plot shows the clustered expression levels of selected genes in ILCs compared to T cells isolated from lymph in steady state. **e** Genes altered in all migratory cells (CD103^+^ DCs, CD103^+^ CD11b^+^ DCs, T cells and ILCs) isolated from lymph compared to the lamina propria, in steady state (fold change >2 and adjusted *p* value < 0.01) were identified and expression levels of genes for ILCs and T cells shown in graph. **f** Transcription levels of migration-associated genes in ILCs isolated from lymph compared to the lamina propria in steady state are displayed as FPKMs. **g** Cells from lymph were isolated by thoracic duct cannulation of C57BL/6 mice that previously underwent mesenteric lymphadenectomy. A representative FACS plot of CD62L and T-bet staining on ILCs in lymph is displayed. FACS Plot is representative for two independent experiments (*n* = 2). **h** At laparotomy, MLNs of Kaede mice were exposed to low-intensity violet light. Small intestinal (sMLN) or colonic (cMLN) draining lymph nodes were removed either immediately or after 24, 48 or 72 h. Isolated cells were stained with antibodies for the analysis by flow cytometry and graphs display the percentages of CD69 expression on ILCs. Each dot represents an individual animal with cMLN and sMLNs isolated from the same mouse (*n* = 3–5) of one experiment. **i**–**j** Transcription levels of selected significantly altered genes in ILCs isolated from lymph compared to the lamina propria in steady state are displayed as FPKMs.

To better characterise migratory ILCs we assessed their transcriptomic profile, by global gene expression analysis, using total ILCs from the lamina propria and total migratory ILCs from intestine-derived lymph. Lymph samples were obtained both from animals in the steady state and after STM infection. The ILCs were compared with CD4^+^ T cells from lymph and lamina propria, collected from the same steady state mice (sort purity shown in Fig. S2A, B). Principal component analysis and unsupervised clustering of the RNA sequencing data revealed that migratory ILCs cluster tightly with tissue-resident ILCs in steady state and are distinct from T cells (Fig. 3a, b). In the unsupervised heatmap of genes that are differentially expressed in any combination (Fig. 3b), clusters of genes associated with particular cells and conditions can be seen. Cluster I is T-cell-specific. Cluster II contains genes that are expressed at higher levels in the lymph. Cluster III is upregulated in T cells and ILCs in the LP. Cluster IV is upregulated in all ILCs, compared to T cells. Similar expression levels of *Id2*, *Zbtb16* and *Gata3*, all key transcription factors for ILC development,^1^ were detected in both migratory and tissue-resident ILCs (Fig. 3c). Consistent with the protein data in Fig. 1, we detected low expression levels of *Rorc* in migratory ILCs compared to tissue-resident ILCs whereas there was no difference in the expression levels of T-bet (Fig. 3c). To investigate whether the migratory ILC population was distinct from T cells, the most likely contaminant, we examined expression levels of genes characteristic of intestinal ILCs. As expected, lymph ILCs show higher expression levels of *Rxrg*, *Cxcr6*, *Gata3*, *Id2*, *Ahr, Rora, Espas1,*^29^ and express low levels of T cell associated transcripts *Cd3e Cd3g*, and *Cd4* (Fig. 3d).

### Migratory ILCs express genes involved in active migration

To assess whether the migratory ILCs in lymph actively migrate from the intestine to the MLNs, we compared tissue-derived lymph cells with their tissue-resident counterparts. We aimed to identify a migratory signature shared by T cells and ILCs. For this analysis, we compared the global transcriptomic profile of ILCs and T cells with similar profiles generated from CD103^+^ DCs and CD103^+^ CD11b^+^ intestinal lymph DCs (Fig. S2C). The DCs are used as a positive control as their active migratory behaviour is well described.^21,26,30^ Interestingly, all cell types share 32 genes that are significantly altered in the migratory population compared to their intestinal tissue-resident counterparts (Fig. 3e and DCs in Fig. S2C). All these genes have similar patterns of expression in T cells and ILCs. We observed several genes known to be associated with regulation of cell migration or motility such as *Rasgrp2,*^31^ *Satb1,*^32^ *S1pr1, S1pr4*^33–35^ and *Sell* (CD62L)^36^ to be commonly upregulated in ILCs, T cells. and in DCs isolated from lymph (Fig. 3e and DCs in S2D). Additionally, comparison of migratory lymph ILCs with ILCs from the lamina propria, also revealed *CCR7* and *CCR10* to be highly expressed in lymph ILCs, whereas lymph ILCs display low expression of *Cd69, Cxcr6, Ccrl2 and Ccr8* (Fig. 3f). We validated transcriptome results with protein staining; we detected CD62L-positive ILCs in intestine-derived steady state lymph (Fig. 3g). We also detected positive protein staining for Ly6d and Ki-67 that correlated with high levels of gene expression on intestine-derived lymph ILCs (Fig. S2E, F). Furthermore, by photoconverting the MLNs of Kaede mice, we detected that recently migrated ILCs lack CD69 (Kaede green cells), whereas up to 20% of tissue-resident ILCs (Kaede red) are CD69 positive (Fig. 3h). Comparisons between the transcriptomes of steady state lymph and tissue-resident ILCs provide clues to the functions of these migratory ILCs. For instance, migratory ILCs express higher levels of *Pdcd1*, *Tigit* and *Cd1d1* (Fig. 3i) and of the genes encoding cytokine receptors IL-12R and IL-17R (Fig. 3j). Thus, migratory ILCs may be poised to respond to T-cell cytokines and influence T-cell responses in the draining lymph node.

### After infection, migratory ILCs express interferon response genes

**Fig. 4:**
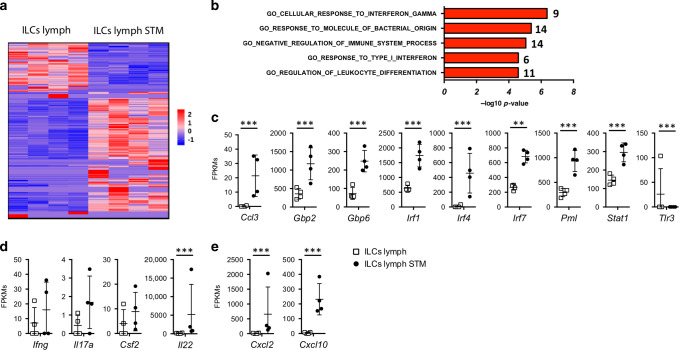
Migratory ILCs express Interferon response genes and change their transcriptional profile. **a** Heatmap is showing the significantly different genes (fold change >2 and adjusted *p* value < 0.01) between migratory ILCs isolated from lymph in steady state and STM-infected mice. Genes (*y*-axis) and samples (*x*-axis) have been clustered using spearman distances and UPGMA agglomeration. **b** The top altered pathways in lymph ILCs after *Salmonella* infection compared to steady state migratory ILCs are shown. **c** Significantly altered genes in STM-infected ILCs that mapped to the cellular response to interferon gamma pathway are displayed as FPKMs. **d** Selected cytokines and chemokines are displayed as FPKMs. **e** Selected genes in migratory ILCs in lymph of STM-infected mice compared to steady state are shown.

To understand how migratory ILCs change during immune responses, we compared migratory ILCs from STM-infected mice to steady state migratory ILCs. 135 genes were significantly upregulated and 77 genes downregulated in STM*-*infected lymph ILCs (Fig. 4a). The complete list of all differentially expressed genes is shown in Fig. S3A. Pathway analysis identified that migratory ILC genes altered during STM infection are commonly associated with interferon signalling (Fig. 4b). After STM infection migratory ILCs express significantly higher levels of *Ccl3, Gbp2, Gbp6, Irf1, Irf4, Irf7, Pml* and *Stat1* (Fig. 4c), all of which are regulated in response to interferon gamma. Thus, intestinal migratory ILCs appear able to respond directly to cytokines. Because RORyt^+^ T-bet^+^ ILCs increased in the lymph of STM-infected mice (Fig. 2b), we assessed expression of ILC1- and ILC3-associated cytokine genes. This revealed increased transcript levels of IL-22 in lymph ILCs after STM infection (Fig. 4d). Furthermore, expression of chemokine genes *Ccl3*, *Cxcl2* and *Cxcl10* also significantly increased in migratory ILCs after STM infection (Fig. 4c, e). Thus, after infection, ILCs show a diverse transcriptomic profile, characterised by a strong response to interferon signalling. Consequently, intestinal ILCs may release chemokines to recruit immune cells, and also produce cytokines to shape adaptive immune responses.

### After STM infection migratory ILCs are activated, and display increased IFNγ production

**Fig. 5:**
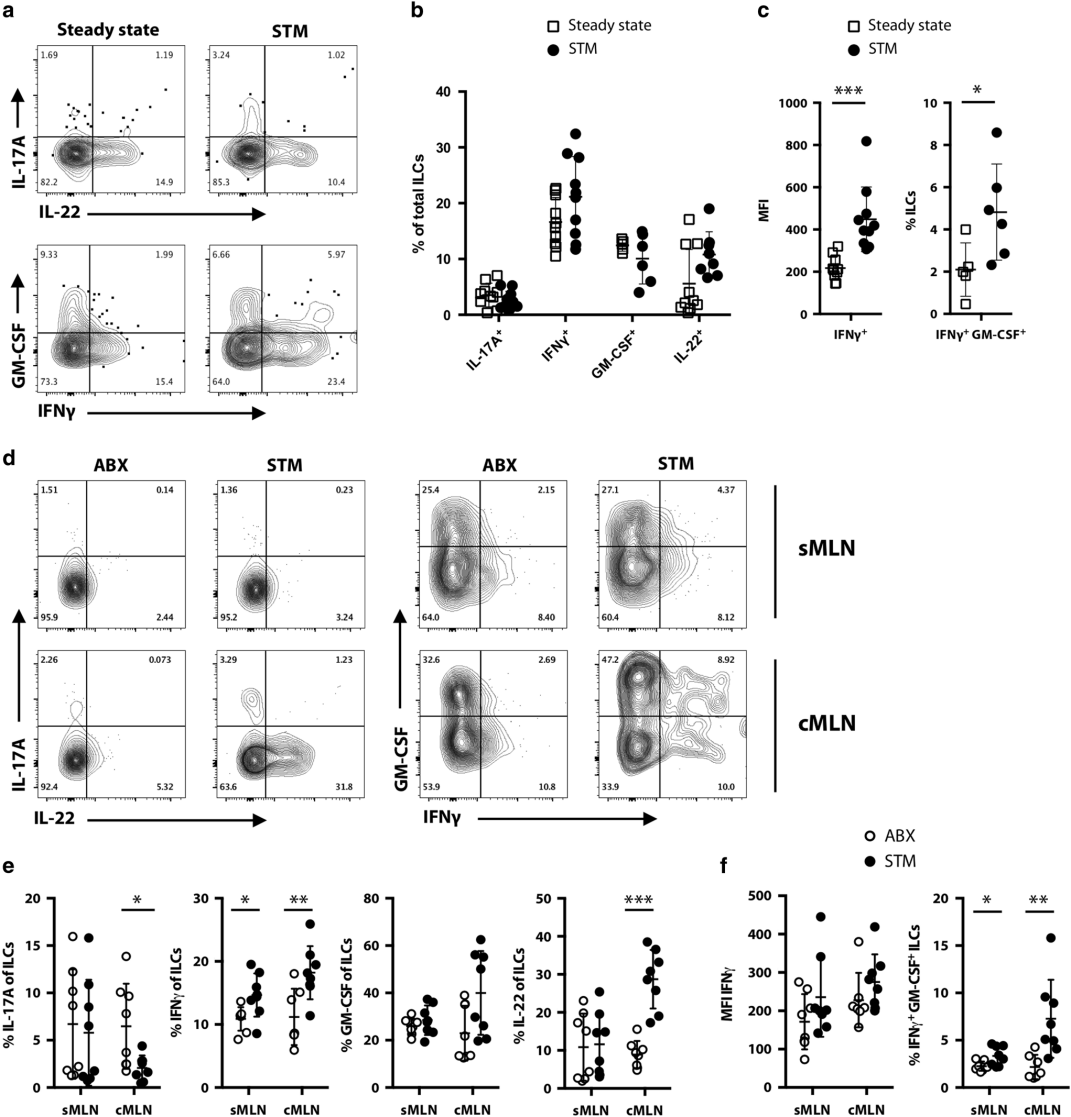
Migratory ILCs display an activated phenotype and display increased IFNγ production during STM infection. **a** C57BL/6 mice that previously underwent mesenteric lymphadenectomy received streptomycin (ABX) and were infected after 24 h with STM. Migratory cells were collected by thoracic duct cannulations on day 2 post infection. ILCs were analysed for the expression of IL-17A, IFNγ, IL-22 and GM-CSF. Cytokine expression is compared to migratory ILCs in the steady state. Representative FACS plots and (**b**) a graph displaying the percentages of cytokine expressing ILCs are shown. **c** Mean fluorescent intensity (MFI) of IFNγ and percentages of IFNγ GM-CSF co-expressing ILCs in pseudo-afferent lymph of ILCs in steady state and *Salmonella* infected mice is displayed. Each dot represents an individual animal and data are from at least two independent experiments (*n* = 5–10). **d** C57BL/6 mice received Streptomycin (ABX) and were infected after 24 h with *S*. Typhimurium (STM). Control mice received Streptomycin only. Cells from the sMLN and cMLN were isolated 72 h post STM infection. ILCs in the MLNs were assessed for expression of the cytokines IL-17A, IFNγ, IL-22 and GM-CSF. Representative FACS plots and (**e**) graphs display the percentages of cytokine expressing ILCs in the sMLN and cMLN. **f** Mean fluorescence intensity (MFI) of IFNγ and percentages of IFNγ GM-CSF co-expressing ILCs in MLN ILCs in ABX control and STM-infected mice is displayed. Data from two independent experiments are shown with each dot representing an individual animal (*n* = 8).

The changes in gene expression after STM infection indicate activation of migratory ILCs. We therefore looked for corresponding changes in protein expression indicative of ILC activation. As transcripts encoding IL-22 were increased in lymph ILCs during infection, we assessed the cytokine profile of migratory ILCs. Although there was no difference in the percentages of ILCs expressing IL-17A, IFNγ, GM-CSF or IL-22 between ILCs from steady state and STM-infected mice, we identified increased expression of IFNγ (assessed by analysing the MFI) after STM infection. During STM infection we also observed an increased proportion of IFNγ/GM-CSF co-expressing lymph-migratory ILCs (Fig. 5a–c). We observed corresponding changes in MLN ILCs after STM infection. The cMLN of STM-infected mice contained a lower frequency of IL-17A producing ILCs, and increased proportions of IL-22 expressing ILCs. We also detected an increased frequency of IFNγ-producing ILCs in both the sMLN and the cMLN (Fig. 5d, e). Unlike in the migratory ILCs, we do not detect an increase in the MFI for IFNγ in MLNs after STM infection unless we pre-gate on IFNγ^+^ ILCs (Fig. 5f and S3B, C). Interestingly, corresponding to the changed cytokine profile detected in lymph, a population of IFNγ GM-CSF double-producing ILCs is found in the cMLN of infected mice (Fig. 5f). These data indicate that ILCs migrating from an inflamed environment, such as in the colonic LP after STM infection, show an activated phenotype and produce cytokines in the draining LNs. Thus, a population of intestinal ILCs can respond to local infection, migrate to the draining lymph node, and help to establish the cytokine environment needed to successfully induce adaptive immunity.

## Discussion

Intestinal ILCs play important roles in regulating tissue homoeostasis and defence against pathogens. Their dynamics in lymph nodes are less well understood. Here we show that all subsets of helper-like ILCs migrate in intestinal lymph. Migratory intestinal ILCs derive from both the colon and the small intestine and express molecules required for migration (CCR7, CD62L, S1P1Rs). After STM infection populations of lymph-migratory ILCs display increased expression of RORyt^+^ and display elevated levels of IFNγ. These changes are reflected in ILCs in colon-draining MLN, indicating that tissue-derived ILCs contribute to shaping the LN environment.

As reported,^6^ we also find that most intestinal ILCs are tissue-resident; only a small fraction migrate into the lymph. Previously it has not been possible to detect ILC migration from tissue to LNs unless the ILCs return to the circulation. Thus, ILC movements in and out of MLNs have not previously been described. Interestingly, while the majority of ILCs in the MLNs are ILC3s, these form only a small fraction of lymph-migratory ILCs in the steady state (Fig. 1h). ILC3s in MLNs are long-lived cells^37^ and our data indicate that MLN ILC3 may be replenished by tissue-derived ILCs relatively slowly compared to ILC1 and ILC2. Due to small numbers of ILCs in the MLNs, migration of a small fraction of intestinal ILCs still replenishes 10% of MLN ILCs within 48 h (Fig. 1k).

In MLN-derived lymph, the majority of ILCs are ILC1s (Fig. 1h), consistent with previous observations.^6,38^ Thus, a population of ILC1s, like NK cells,^39^ circulates between LNs.

Analysis of the transcriptomes of migratory ILCs and tissue ILCs, revealed a migratory signature shared with both DCs and CD4^+^ T cells (Fig. 3e). Our intestinal migratory ILCs, express S1P receptors, CCR7, CD62L and lack CD69. An ILC2 population that traffics from the intestine to the lung during helminth infections displays similar properties, expressing S1PRs but not CD69.^34^ Thus, all ILCs may depend on similar receptors for their migration. Migration requires a co-ordinated and complex set of changes in gene expression, and only a small proportion of ILCs undergo these changes, even after STM infection; these will be an important focus of future work.

After infection with STM migratory ILCs appear to respond to interferons (Fig. 4), and a population of migratory RORyt^+^ T-bet^+^ ILCs increases (Fig. 2). Infection induces increased ILC production of IFNγ, and some begin to co-express GM-CSF (Fig. 5). This co-expression of IFNγ and GM-CSF in migratory ILCs correlates with the increase in the proportion of RORyt^+^ T-bet^+^ ILCs. This RORyt^+^ T-bet^+^ ILC population is an important early source of IFNγ in the lamina propria during STM infection,^28^ and it would be interesting to examine whether this may also contribute to the GM-CSF produced in the intestine after STM infection. Here we now show that RORyt^+^ T-bet^+^ ILCs may also be crucial in the draining LNs early during the immune response. Indeed, consistent with our lymph data, we detect a parallel accumulation of RORyt^+^ T-bet^+^ ILCs, and changes in the cytokine profile of ILCs in the MLNs during STM infection. It is possible, and perhaps likely, that migratory ILC1s and/or MLN-tissue-resident ILCs might also contribute to the cytokine profile observed in cMLN ILCs in response to STM infections (Figs. 3 and 5).

During Th1 priming, IFNγ in the lymph node drives the differentiation of Th1 cells. NK cells are an early source of this IFNγ.^40^ We now show that migratory ILCs are also an early source of IFNγ in the LN. Similarly, the GM-CSF released by ILCs locally in the interfollicular area might further support the anti-STM response by enhancing T cell priming by DCs,^41^ and by recruiting other immune cells, including neutrophils, into the MLN.^42,43^ The specific location of ILCs in the LN interfollicular area, close to DCs, T cells and B cells,^11^ makes them credible as influencers of adaptive immune responses. Furthermore, migratory ILCs express increased levels of *Cxcl10* after STM infection so may actively contribute to intranodal positioning of CXCR3-expressing T cells^44^ and recruitment of NK cells^40^ into the MLN to enhance Th1 responses. Thus, migratory ILCs may contribute to T-cell differentiation by augmenting critical cytokine levels early during the response in the MLN, and by contributing to recruitment of both cytokine-producing and responder cells. However, many questions still remain, including: what signals drive ILC to exit the tissue; how is migration regulated during inflammation; and in the absence of migratory ILCs, are adaptive immune responses less efficiently induced, or delayed?

Our data provide a detailed characterisation of migratory intestinal ILCs. We show that migratory ILCs supplement ILCs in lymph nodes, and that infection activates migratory ILCs. These data indicate the potential for crosstalk between migratory ILCs and other immune cells in the LN, which may contribute to the early events that shape adaptive immune responses.

## Material and methods

### Mice

C57BL/6 mice were purchased from Envigo Laboratories at 5–8 weeks of age and maintained in individually ventilated cages (IVC) at the Central Research Facility, Glasgow, UK. Id2^+/GFP^ and *Rorc(γt)*^+/GFP^mice were bred at the Veterinary Research Facility, Glasgow, UK. Id2^+/GFP^ mice were originally generated by Gabrielle Belz (Walter and Eliza Hall Institute of Medical Research, Australia)^23^ and *Rorc(γt)*^+/GFP^ mice were generated by Gerard Eberl (Institut Pasteur, France).^45^ Kaede mice (originally from Kenji Kabashima)^46^ were bred at the Central Research Facility, Glasgow, UK. All mice were bred and housed under specific pathogen free conditions. All procedures were approved by the local ethical committee and carried out under licenses issued by the UK Home Office.

### Surgical procedures

#### Kaede photoconversion

As previously reported,^26^ for the photoconversion of the Kaede green protein in the MLN, the lymph nodes of *Kaede* transgenic mice were exposed to low-intensity violet light (395 nm UV LED) for 6 min after laparotomy. The MLNs were exposed to the violet light whilst protecting the surrounding tissue from light using aluminium foil.

#### Mesenteric lymphadenectomy and thoracic duct cannulations

Mesenteric lymphadenectomy and thoracic duct cannulations were performed according to published protocols.^47^ Mesenteric lymphadenectomy was performed on 5–9-week old male mice. Thereby, the complete MLN chain was removed by blunt dissection after laparotomy. Mice were allowed to recover for at least 6 weeks in IVCs before the thoracic duct was cannulated. Lymph was collected on ice for 18–24 h in PBS with 20 U/ml of heparin (Wockhardt).

#### Cell isolations from tissues

For the isolation of cells from the small intestinal and colonic lamina propria, the tissues were cut longitudinally and into sections following the removal of fat. Peyer’s Patches (PPs) were removed from SI samples. The tissues were incubated for two rounds in HBSS with 2 mM EDTA at 37 °C, for 20 min for the SI or 15 min for the colon, in a shaking incubator. The SI was digested in complete RPMI supplemented with 1 mg/ml Collagenase VIII (Sigma) at 37 °C for ~15 min. The colon was digested in complete RPMI containing 0.85 mg/ml Collagenase VIII (Sigma), 1.25 mg/ml Collagenase D (Roche, Switzerland), 1 mg/ml Dispase (Gibco) and 30 μg/ml DNAse (Roche) at 37 °C for ~40 min. After the digest, cells were passed through a 100 and 40 μm cell strainers.

LNs were cut into smaller pieces and digested for 20 min at 37 °C in RPMI supplemented with 0.075 mg/ml DNAseI (Roche) and 0.75 mg/ml Collagenase-Dispase (Roche). After the digest, cells were passed through a 40 μm cell strainer.

Lymph cells were passed through a 40 μm cell strainer.

#### *S*. Typhimurium infections

Mice received 20 mg streptomycin (Sigma) in 100 μl PBS via oral gavage 24 h before infection with *Salmonella enterica serovar* Typhimurium (*S*. Typhimurium) SL1344. *S*. Typhimurium was grown in luria broth (LB) at 37 °C for 5 h at 180 rpm. After incubation, the cultures were diluted 1:2 in LB and further kept in a static incubator overnight. The following day, bacterial cell density was adjusted to an OD600 of 0.75. Intact or lymphadenctomised mice received 5–8 × 10^7^ colony forming units bacteria in 100 μl PBS via oral gavage.

#### Cell stimulation for cytokine analysis

For the analysis of cytokine expression, cells were stimulated with the eBioscience cell stimulation cocktail including protein transport inhibitors for 4 h at 37 °C according to the manufacturer’s instructions (Thermofisher scientific).

#### FACS antibody staining

Antibody staining for extracellular molecules was performed at 4 °C for 30 min. Intracellular staining was performed at room temperature for 1 h using the FoxP3 fixation and permeabilisation kit (Thermofisher scientific) according to the manufacturer’s instructions. The following antibodies were purchased from Biolegend: B220 (RA3-6B2), CCR6 (29-2L17), CD11b (M1/70), CD11c (N418), CD45 (30-F11), I-Ab (M5/114.15.2), IL-7Rα (A7R34), CD44 (IM7), CD64 (X54-5/7.1), CD4 (GK1.5), CD8α (53-6.7), CD19 (6D5), CD103 (2E7), IL-17A (TC11-18H10.1), CD5 (53-7.3), CD3 (17A2), CD44 (IM7), CD25 (PC61), CD62L (MEL-14), CD69 (H1.2F3). Ly6D (49-H4), GM-CSF (MP1-22E9), IL-22 (Poly5164), Thy-1.2 (53-2.1). Other antibodies were purchased from BD Biosciences: IFNγ (XMG1.2), CD11c (HL3) or from Thermofisher scientific: CD3ε (145-2C11), GATA3 (TWAJ), RORγt (AFKJS-9), T-bet (eBio4B10), Ki-67 (SolA15).

The eFluor 780 viability dye (Thermofisher scientific) was added to identify live cells. Samples used for cell sorting were incubated with the dead live dye 7AAD (Biolegend). Flow cytometry analyses were performed on LSR II, LSR Fortessa analyser, FACSAria IIu or FACSAria III cell sorter (all BD Biosciences) using the FACSDiva6.2 software (BD Biosciences), or sorted by fluorescence-activated cell sorting using the FACSAria IIu or FACSAria III cell sorter. Data were subsequently analysed with the FlowJo software (Tree Star).

#### RNA sequencing

Cells were isolated from C57BL/6 mice that previously underwent complete mesenteric lymphadenectomy. ILCs were identified as live, single, CD45^+^ IL-7Rα^+^ lineage^−^ (CD3, CD5, CD11b, CD11c, B220) cells and T cells as live, single, CD45^+^ CD11b^−^ CD11c^−^ B220^−^ CD3^+^ CD5^+^ CD4^+^ CD44^high^ CD25- cells. Cells for RNA seq were purified by flow cytometric sorting, collected into 350 μl RLT plus buffer (Qiagen) and stored at −80 °C until further processing. Samples were sorted in quadruplicates and isolated cell types included migratory ILCs (*n* = 15) and T cells (*n* = 8) from afferent lymph of thoracic duct cannulated mice in steady state, ILCs (*n* = 4) and T cells (*n* = 4) from the colonic and small intestinal lamina propria at similar proportions, and migratory ILCs (*n* = 25) from afferent lymph of *Salmonella* infected mice that underwent thoracic duct cannulation. RNA was isolated according to the manufacturer’s protocol (RNAeasy plus micro kit, Qiagen).

CD11b^+^ CD103^−^ and CD11b^+^ CD103^+^ DCs (live, single, CD45^+^, CD64^−^ MHCII^high^ CD11c^+^) were isolated from the small intestinal lamina propria of C57BL/6 mice after removal of PPs. The migratory DC counterparts were isolated by thoracic duct cannulations of mice that previously underwent complete mesenteric lymphadenectomy. RNA was isolated according to the manufacturer’s protocol (RNAeasy kit, Qiagen).

The ILC and T-cell sequencing libraries were prepared according to published protocols,^48^ using a TAKARA SMART-seq v4 ultra low input library preparation kit with 15 cycles of amplification, and were sequenced to a mean depth of 5.1 million read pairs per sample using an Illumina HiSeq 2500. The DC sequencing libraries were prepared using a TAKARA SMART-seq v4 ultra low input library preparation kit, and were sequenced to a mean depth of 37.5 million read pairs per sample using an Illumina HiSeq 4000. The read length for both libraries was 75 bp. A high read quality (mean score above 28) in all samples was confirmed using FastQC. Reads were aligned to the mouse genome (GRCm38) using Star (v2.6.0c)^49^ with the parameters –quantMode GeneCounts, –outFilterMultimapNmax 1 and –outFilterMatchNmin 35. The Star index was generated with the parameters –sjdbGTFfile (transcriptome version 93) and –sjdbOverhang 74. The mean alignment rate was above 85% per sample. Read counting was performed by star using unstranded counting for both datasets. For both datasets normalised counts and differential expression values were generated using DESeq2^50^ under default settings. Genes with an adjusted *p* value of less than 0.01 and an absolute fold change of greater than 2 were considered significant.

The RNA-seq data, available at the NCBI GEO database (GSE160156), was explored using Searchlight 2 (v2.0.0a)^51^ and R (version 3.4.4). Specifically, enrichment of gene ontologies was generated using a standard hypergeometric test with Benjamini–Hochberg multisample correction, and the gene-set database Gene Ontologies Molecular Function (GO_MF) as downloaded from MsigDB collections.^52^ Principal Component analysis was performed on *Z*-score transformed expression data, using R. Heatmap clustering was performed using the R package fastcluster using spearman distances, UPGMA agglomeration and mean reordering.

### Statistical analysis

Prism Software (GraphPad) was used to perform statistical analysis. Data are presented as mean ± SD. For comparison of means between two groups, the data were analysed using a Mann–Whitney *U*-test. For comparisons involving more than two data sets, a 2-Way ANOVA was used. A Bonferroni post-test was applied to correct for multiple comparisons. *P* values of <0.05 were considered significant, *p* values *<0.05, **<0.01, ***<0.001.

## Supplementary information

Supplementay Figures
