# Supplementary material for: Intestinal-derived ILCs migrating in lymph increase IFNγ production in response to Salmonella Typhimurium infection
Source: Mucosal Immunol. 2021 Jan 7;14(3):717–27. doi: 10.1038/s41385-020-00366-3 (PMC8075955; doi:10.1038/s41385-020-00366-3)
Supplement: Supplementary file 1 — Supplementay Figures [file 41385_2020_366_MOESM1_ESM.pdf]

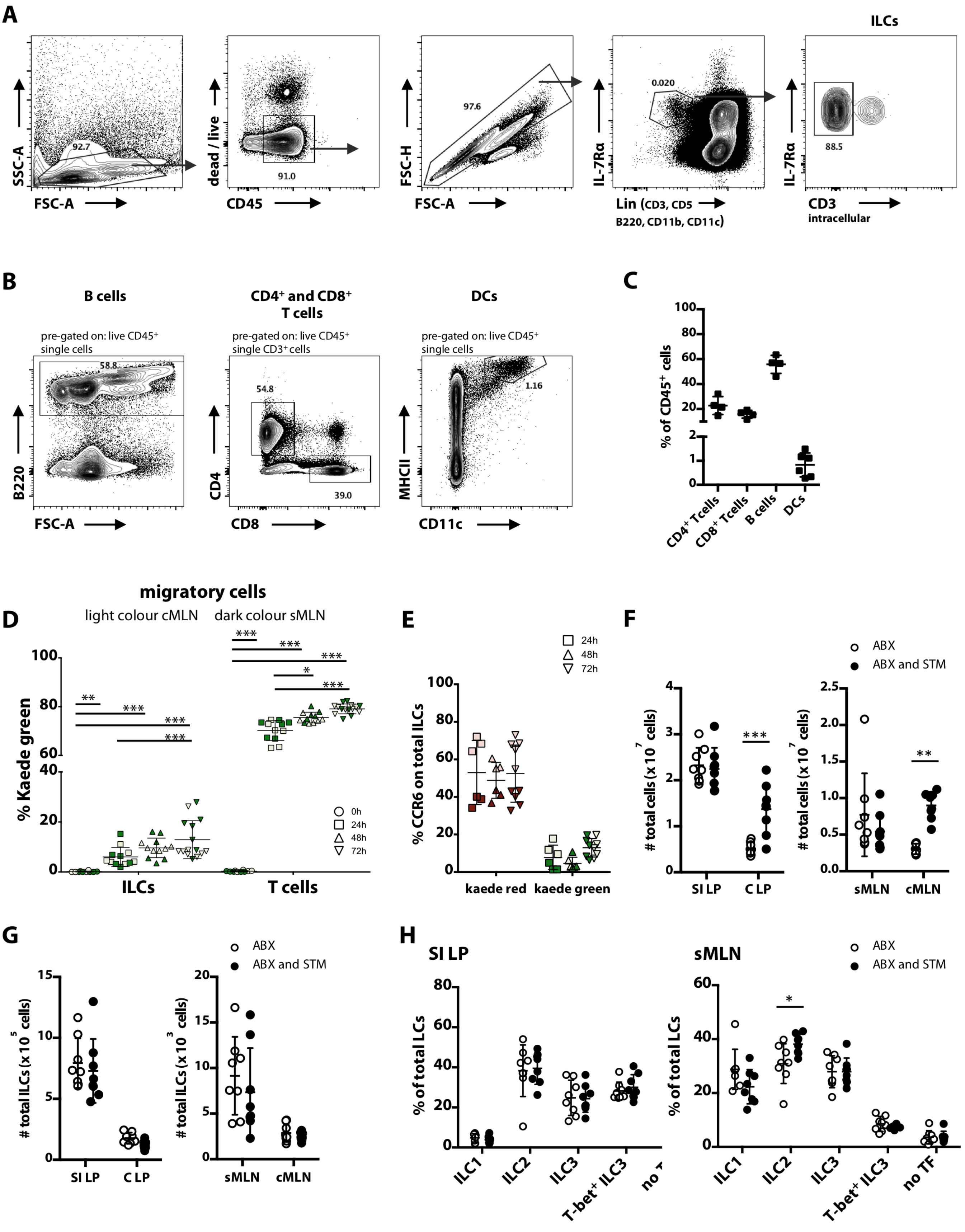

Supplementary Figure 1

Figure S1: **Characterisation of intestinal lymph ILCs**

Cells from lymph were isolated by thoracic duct cannulations of C57BL/6 mice that previously underwent mesenteric lymphadenectomy. Migratory cells were collected for 18-24h and stained with antibodies for phenotypic analysis by flow cytometry. Representative FACS plots for gating strategies of (**A**) ILCs (live single $CD45^+$ IL-$7R\alpha^+$ $CD3^-$ $CD5^-$ $CD11b^-$ $CD11c^-$ $B220^-$ and intracellular $CD3^-$ cells, (**B**) B cells (live single $CD45^+B220^+$ cells), T cells (live, single, $CD45^+$ $CD3^+$ $CD5^+$ $CD4^+$ or $CD8^+$ cells) and DCs (live, single, $CD45^+$ $MHCII^+$ $CD11c^+$ cells). **(C)** Tissue derived lymph samples were stained for B cells, T cells and DCs and the composition displayed in a graph. Data from three independent experiments are shown. Each symbol represents an individual animal (n = 4-6). (**D**) Lymph nodes of Kaede mice were exposed to violet light at laparotomy. Immediately, or after 24, 48, or 72h, sMLN and cMLN were collected separately and the proportions of Kaede green only ILCs (live single $CD45^+$ IL-$7R\alpha^+$ $CD3^-$ $CD5^-$ $CD11b^-$ $CD11c^-$ $B220^-$ Kaede $green^+$ Kaede red^- cells) and T cells (live, single, $CD45^+$ $CD3^+$ $CD5^+$ Kaede $green^+$ Kaede red^- cells) were assessed. **(E)** Graph shows the expression of CCR6 on photoconverted Kaede red and non-converted Kaede green ILCs in sMLNs (dark colour) or cMLNs (light colour) up to 72 h post laparotomy. Each dot represents an individual animal (n = 3-5) of one experiment. In **(F-H)** C57BL/6 mice received streptomycin (ABX) and were infected after 24h with STM. Control mice received streptomycin only. Each dot represents an individual animal (n = 8) from 4 independent experiments. **(F)** Cells from the small intestinal and colonic LP and the sMLNs and cMLN were isolated three days after STM infection. Graphs display cell numbers isolated in the LP and MLN of STM infected and control mice. Graphs display **(G)** total cell numbers of ILCs and cell numbers of ILC subsets in the **(H)** lamina propria of the SI and sMLNs of control and STM infected mice.

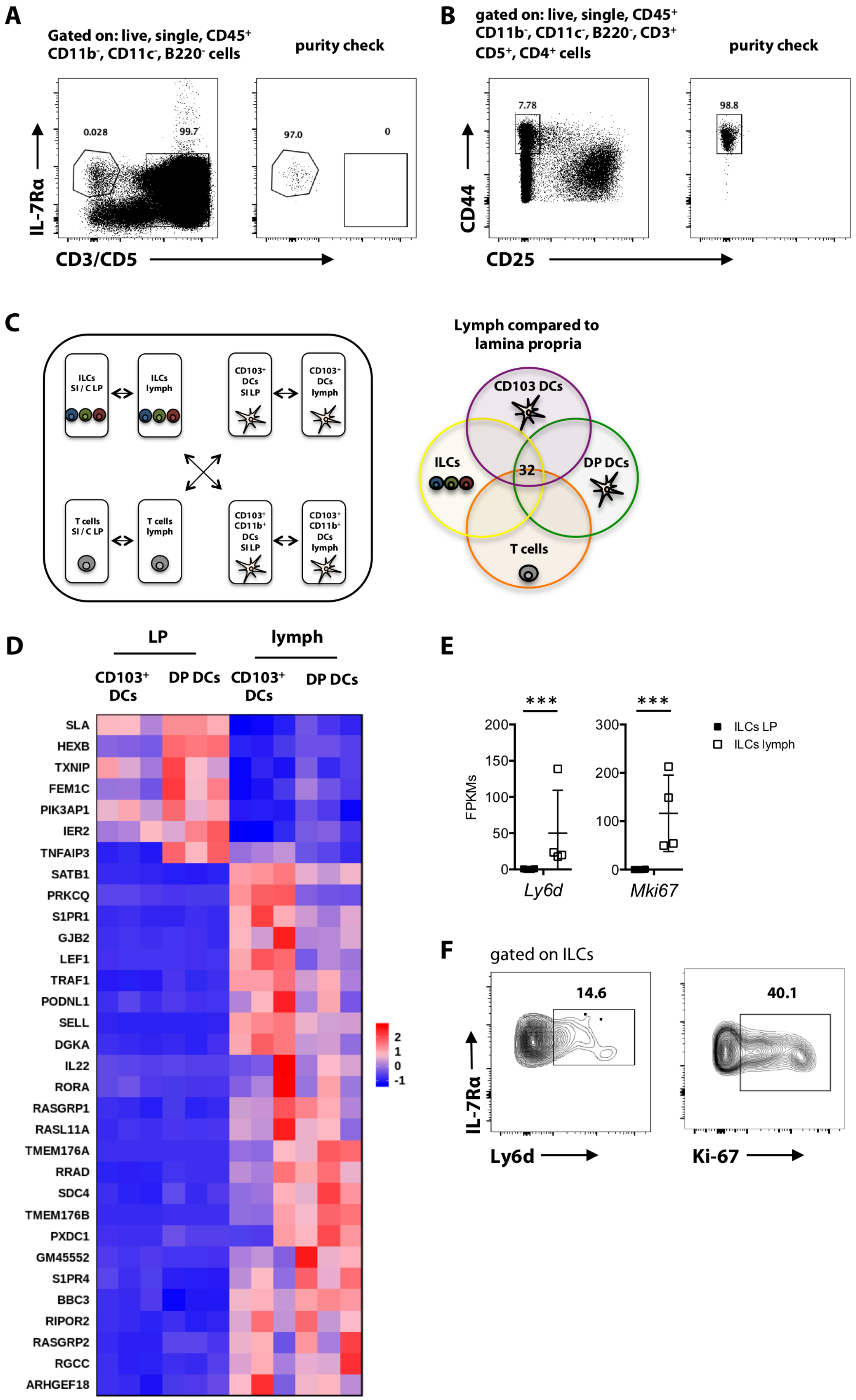

Supplementary Figure 2

Figure S2: **Migratory cells in intestinal lymph display a shared migratory signature**

Migratory cells were isolated from C57BL/6 mice that previously underwent mesenteric lymphadenectomy. Lymph was isolated by thoracic duct cannulations of untreated (steady state) or STM infected mice. Cells were isolated and sort purified by flow cytometry and the sort purity checked pre and post sort. Representative FACS plots of the lymph samples and purity check of **(A)** ILCs and **(B)** T cells in steady state are shown. FACS plots are representative for 4-5 independent experiments for each, steady state and *STM* infection. Sort purity of sequenced samples was > 95%. **(C)** The transcriptomic signature of migratory cells isolated from the thoracic duct are compared to the corresponding cells isolated from the lamina propria. Genes commonly altered in ILCs, T cells and DC subsets ($CD103^+$ $CD11b^-$ and $CD103^+$ $CD11b^+$) were assessed. Diagram shows 32 genes that are significantly altered in all four cell types when cells enter the lymphatic system compared to the LP. **(D)** Heatmap shows the significantly 32 upregulated or downregulated genes between migratory cells isolated from the thoracic duct compared to the lamina propria in steady state of $CD103^+$ $CD11b^-$ DCs and $CD103^+$ $CD11b^+$ (double positive) DCs. The levels of expression are row scaled into z-scores. **(E)** The transcriptional profile was assessed by performing RNA-sequencing and compared to ILCs isolated from the lamina propria. FPKMs of selected genes in lymph ILCs compared to the lamina propria in steady state are shown. **(F)** ILCs isolated from lymph were analysed for protein expression of the selected genes by flow cytometry. Representative FACS plots of at least 2 individual lymph samples are shown.

A **ILCs lymph steady state vs STM**

downregulated	upregulated
STAU2	TGOLN1
GM37101	STAT1
KDELC2	GNA13
TBC1D30	TAP1
TLR3	TGTP2
B3GNT9	TPR
CLIP3	MAPKAPK2
GM47015	TCP11L2
DCBLD2	G6PDX
GM32856	IRS2
UNC5B	APLP2
MTUS1	F2R
UTP14B	NEK9
4933404O12RIK	TRP53INP1
SPDL1	PPP1R15A
TMEM30B	FURIN
PLEKHD1	AP2A1
FAM212B	TMEM123
ACTA2	OSBPL9
BCAM	TBC1D9B
TDRP	OXR1
SCARB1	SIPA1L1
TGFBR3	ZFP36
FGF13	TNFAIP3
ACP2	IL4RA
ACAD10	MXD1
SLC2A4RG-PS	IGTP
DNAJC27	IRF7
FRY	SCPEP1
METTL15	TXNDC11
KIF3C	SBNO1
LRSAM1	SAT1
TTN	P2RY10
GRAMD1C	PAPD4
CD99L2	CSRNP1
GM11110	CREM
GM37352	NUS1
TMIGD1	IRF1
MILR1	RNF125
SLC12A9	EPB41
D3ERTD751E	GVIN1
SYNE2	ECE1
ADAMTS10	STAT3
AAMDC	ZFP36L1
FLT3L	LY6A
GM16702	GPR18
HIVEP2	ITGAV
COA6	PLSCR1

downregulated	upregulated
KLRG1	ARAP2
CD9	GEM
SELENOH	RTP4
DNAJC15	PML
NMUR1	BRPF1
CD3G	SBNO2
3830406C13RIK	GBP2
UBASH3A	SOCS3
S100A10	DGAT1
CD27	PARP10
TSPAN32	GBP6
RAB37	ISG20
EIF1B	TGTP1
PTPRCAP	SEC63
LGALS1	BHLHE40
ARHGAP4	MARCKSL1
DCTN6	ABCB1A
WBP1	XBP1
CD52	SLC39A11
EMP3	RAMP3
RASGRP2	ST3GAL3
SMPDL3A	IRGM1
HINT1	PDE4B
TMSB4X	SERPINB9
TRAF3IP3	NBN
CNOT6L	GCNT2
HEXB	LILR4B
RPL12	RGS1
GLIPR2	SDC1
	FRMD4B
	SMOX
	TRAC
	LITAF
	GM26809
	CHAD
	SEC24A
	CTLA4
	CD274
	GADD45G
	CDKN1A
	GPR55
	ATF3
	CPE
	CDK14
	TIAM2
	IL22
	CIT
	IRF4

downregulated	upregulated
	CXCL2
	CXCL10
	GM19557
	CCL3
	SCRT1
	CHST1
	FOXP3
	RHOBTB1
	GM12185
	RGMB
	IGHV9-3
	MYOF
	MCPT1
	ZFP442
	RIN2
	THBD
	GM38111
	DACT3
	APH1B
	LRIG1
	ZFP229
	EXTL1
	CCDC116
	ABLIM2
	ADAMTS1
	FZD7
	FGGY
	KCNA2
	DGKH
	DNAH11
	ZFR2
	MAP10
	DRAM1
	SEMA6A
	ATP6V0A1
	SEMA6D
	IGHV1-55
	IGKV5-48
	IGKV8-27

B

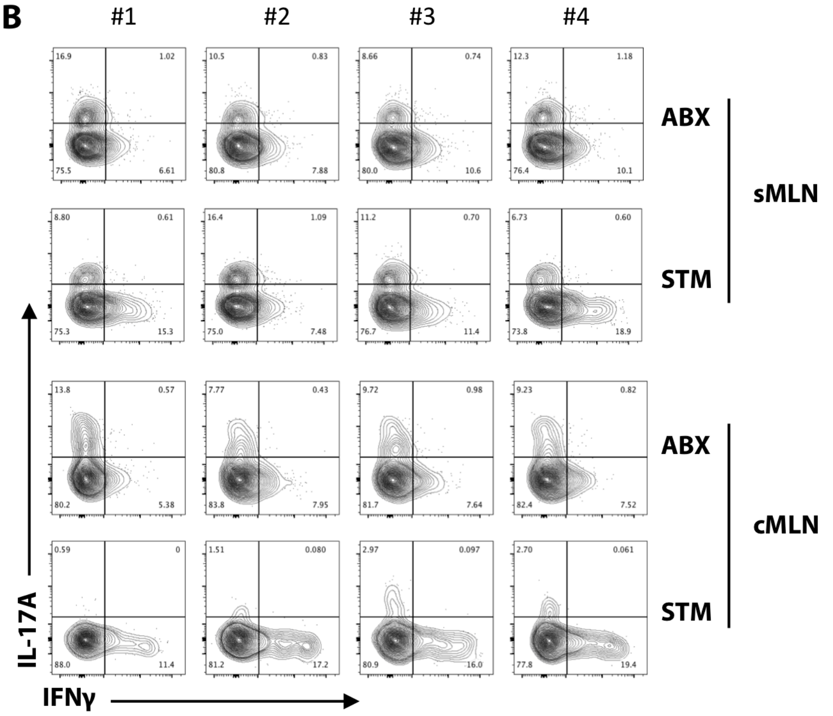

C

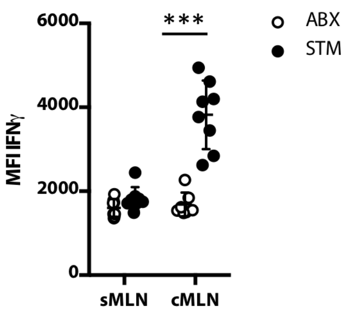

Supplementary Fig 3

Figure S3: **Differentially expressed genes in ILCs after STM infection.**

Migratory ILCs were isolated from C57BL/6 mice that previously underwent mesenteric lymphadenectomy. ILCs were isolated from steady state mice or mice that were infected with STM. The transcriptional profile was assessed by performing RNA-sequencing and **(A)** differentially expressed genes of lymph ILCs after STM compared to lymph ILCs in steady state. **(B)** Intact C57BL/6 mice received Streptomycin (ABX) and were infected after 24h with *S*. Typhimurium (STM). Control mice received Streptomycin only. Cells from the sMLN and cMLN were isolated three days post STM infection. ILCs in the MLNs were assessed for the cytokines IL-17A and IFNγ. FACS plots display staining in individual animals (#1-#4) in control and STM infected mice of one representative experiment. **(C)** Mean fluorescent intensity (MFI) of IFNγ gated on IFNγ^{+} ILCs in the sMLN and cMLN are displayed. Each dot represents an individual animal (n = 8) from two independent experiments.
